# Isolated FeN_4_ Sites for Efficient Electrocatalytic CO_2_ Reduction

**DOI:** 10.1002/advs.202001545

**Published:** 2020-07-12

**Authors:** Xiaogang Li, Shibo Xi, Libo Sun, Shuo Dou, Zhenfeng Huang, Tan Su, Xin Wang

**Affiliations:** ^1^ School of Chemical and Biomedical Engineering Nanyang Technological University 62 Nanyang Drive Singapore 637459 Singapore; ^2^ Institute of Chemical and Engineering Sciences A*STAR Singapore 627833 Singapore; ^3^ Laboratory of Theoretical and Computational Chemistry Institute of Theoretical Chemistry Jilin University Changchun 130012 P. R. China

**Keywords:** CO_2_ reduction, confined pyrolysis, electrocatalysis, isolated active sites

## Abstract

The construction of isolated metal sites represents a promising approach for electrocatalyst design toward the efficient electrochemical conversion of carbon dioxide (CO_2_). Herein, Fe‐doped graphitic carbon nitride is rationally prepared by a simple adsorption method and is used as template to construct isolated FeN_4_ sites through a confined pyrolysis strategy, which avoids the agglomeration of metal atoms to particles during the synthesis process and thus provides abundant active sites for the CO_2_ reduction reaction. The isolated FeN_4_ sites lower the energy barrier for the key intermediate in the CO_2_ reduction process, leading to the enhanced selectivity for CO production with a faradaic efficiency of up to 93%.

## Graphical abstract

Isolated FeN_4_ sites in carbon substrate are constructed through a confined pyrolysis strategy with graphitic carbon nitride as the template. The isolated FeN_4_ sites show lower free energy change for the key intermediate in the CO_2_ reduction process, leading to enhanced selectivity for CO production with a faradaic efficiency of up to 93%.

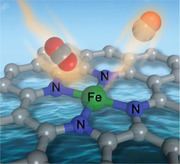

The accumulation of CO_2_ in atmosphere due to the excessive consumption of fossil fuels has posed potential environmental concerns.^[^ ^1^ ^]^ Electrocatalytic CO_2_ reduction offers a promising approach to mitigate CO_2_ levels and convert it to value‐added fuels.^[^ ^2^ ^]^ Many efforts have been dedicated to developing efficient electrocatalysts for CO_2_ reduction. Nevertheless, the low selectivity of the reduction products is still a key obstacle which restricts the further development of CO_2_ conversion technologies.^[^ ^3^ ^]^ Homogeneous catalysts could possess excellent selectivity due to its homogeneously distributed and well‐defined active sites, while its inferior stability and difficulty of separation hinder the further development toward industrial applications.^[^ ^4^ ^]^ In contrast, heterogeneous catalysts could provide stable catalytic performance, while the diverse nature of active sites restricts the selectivity.^[^ ^5^ ^]^ Thus the development of catalysts that combine the advantages of both heterogeneous and homogeneous catalysts is urgently imperative for the efficient conversion of CO_2_.

Single‐atom‐based catalysts provide a great potential to bridge the gap between heterogeneous and homogeneous catalysts.^[^ ^6^ ^]^ Apart from the maximum atom efficiency, single‐atom catalysts afford the isolated and well‐defined active sites confined in the support of inorganic solid material, offering high selectivity and stability toward catalytic reaction.^[^ ^7^ ^]^ Benefiting from the unique structure, the single‐atom catalysts present comparable catalytic activity to that of homogeneous catalysts, meanwhile possessing high recyclability and stability arising from heterogenization. Among the single‐atom catalysts, the isolated metal atoms coordinated with nitrogen (MN_*x*_) in carbon substrates have shown excellent performance in electrocatalysis,^[^ ^8^ ^]^ providing a promising way for electrocatalytic CO_2_ reduction. However, construction of MN_*x*_ sites is still a challenge since isolated active sites are easily agglomerated to particles during the synthesis process, leading to loss of catalytic performance. Recently, isolated NiN_4_ sites have been successfully constructed via a confined pyrolysis strategy, in which Ni‐doped graphitic carbon nitride (g‐C_3_N_4_) acts as the template and nitrogen source during the confined pyrolysis process.^[^ ^9^ ^]^ Considering the synthesis of metal‐doped g‐C_3_N_4_ by pyrolyzing the precursor of g‐C_3_N_4_ and metal salts is uncontrollable, a more simple and universal design of metal‐doped g‐C_3_N_4_, which is the pivotal step in the confined pyrolysis strategy, is still much desirable but remains challenging for the construction of MN_x_ sites.

Herein, we report the construction of isolated FeN_4_ sites in carbon substrates (denoted as FeN_4_/C) by the rational design of Fe‐doped g‐C_3_N_4_. A simple adsorption method was adopted for g‐C_3_N_4_ to trap Fe atoms, making it an excellent template for the formation of FeN_4_ sites. Then the formation of a carbon layer on the surface of Fe‐doped g‐C_3_N_4_ would provide a confined environment to suppress the agglomeration of Fe atoms to particles during the pyrolysis process, thus effectively constructing the isolated active sites. Benefiting from the unique structure and coordination environment, the isolated FeN_4_ sites show high selectivity for the electrocatalytic conversion of CO_2_ to CO, with the highest faradaic efficiency of 93% at −0.6 V versus RHE. The isolated configuration was revealed by spherical aberration correction electron microscopy and extended X‐ray absorption fine structure analysis. The theoretical calculation demonstrates that isolated FeN_4_ sites lower the energy barrier for the formation of COOH*, leading to the enhanced activity for CO production. We believe this study would pave a new avenue for rational design of highly efficient single atom catalysts with abundant active sites.

The morphology of the catalysts was confirmed by transmission electron microscopy (TEM). **Figure** **1**a shows the TEM image of FeN_4_/C, which presents a sheet‐like structure, similar to that of bare N‐doped carbon (denoted as N/C) and g‐C_3_N_4_, as seen in Figure S1 (Supporting Information). No particles are observed from the high‐magnification TEM image for FeN_4_/C, as shown in Figure 1b. The high‐angle annular dark‐field scanning transmission electron microscopy (HAADF‐STEM) image of FeN_4_/C in Figure 1c reveals that isolated bright spots corresponding to Fe atoms are homogeneously dispersed, demonstrating the single‐atom form of Fe in the FeN_4_/C. Energy‐dispersive X‐ray spectroscopy (EDX) mapping analysis in Figure 1d indicates that Fe and N atoms distribute homogeneously in carbon substrate.

**Figure 1:**
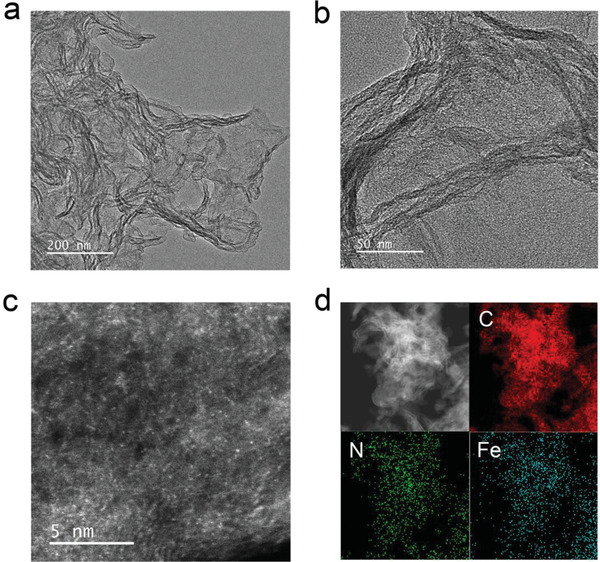
a) Low‐ and b) high‐magnification TEM images of FeN_4_/C. c) HAADF‐STEM image of FeN_4_/C. d) Element mapping image of FeN_4_/C.

The Raman spectrum for FeN_4_/C and N/C in Figure S2 (Supporting Information) shows two distinct peaks at about 1570 and 1330 cm^−1^, which could be assigned to graphitic sp^2^ carbon (G‐band) and disordered sp^3^ carbon (D‐band).^[^ ^10^ ^]^ The ratio of the relative intensity of the D band to the G band presents no much difference for FeN_4_/C and N/C, with an *I*_D_/*I*_G_ value of 1.38 and 1.31, respectively, indicating the similar extent of graphitization and disorder in the catalysts. The X‐ray diffraction (XRD) patterns of FeN_4_/C and N/C in Figure S3 (Supporting Information) both show two typical broad peaks at about 24° and 44°, which could be ascribed to (0 0 2) and (1 0 1) lattice plane of graphite.^[^ ^11^ ^]^ Apparently there is no new crystal phase present in the FeN_4_/C. While without the confinement of the carbon layer during pyrolysis, Fe_3_C and Fe particles would appear in the synthesized catalyst (denoted as Fe/C). As shown in the TEM images in Figure S4 (Supporting Information), the catalyst shows a morphology of particles wrapped with outer carbon layer. XRD pattern of Fe/C in Figure S5 (Supporting Information) presents the distinct peaks corresponding to Fe_3_C (JCPDS Card No. 350772) and Fe (JCPDS Card No. 060696), demonstrating the dominant species in Fe/C are Fe_3_C and metallic Fe. Note that the further additional loading of Fe will also result in the formation of Fe nanoparticles in FeN_4_/C (denote the catalyst as Fe NPs/C). As seen in Figure S6a (Suppoerting Information), the XRD pattern of Fe NPs/C shows a distinct peaks at 44.6°, corresponding to the (1 1 0) lattice planes of metallic Fe (JCPDS Card No. 060696). The TEM image in Figure S6b (Supporting Information) shows the presence of small particles on the Fe NPs/C. The EDX mapping clearly shows the aggregation of Fe atoms to particles, as shown in Figure S6c (Supporting Information). The high resolution TEM (HRTEM) image of Fe NPs/C in Figure S6d (Supporting Information) presents a lattice distance of 0.20 nm, which could be ascribed to the (1 1 0) plane of Fe particles (JCPDS Card No. 060696), agreed well with the XRD result.

Synchrotron‐based X‐ray absorption spectroscopy was further adopted to determine the precise local chemical configuration around isolated Fe sites. **Figure** **2**a presents the Fe K‐edge X‐ray absorption near‐edge structure (XANES) curves of FeN_4_/C, in comparison to Fe NPs/C and Fe foil. It is clearly seen that the absorption‐edge of FeN_4_/C shifts toward higher energy compared with Fe NPs/C and Fe foil, suggesting Fe in FeN_4_/C is in an oxidation state.^[^ ^12^ ^]^ As a comparison, the absorption‐edge of Fe NPs/C is located between the FeN_4_/C and Fe foil, which could be ascribed to the coexistence of oxidized Fe single atoms and metallic Fe particles. The Fe 2p X‐ray photoelectron spectroscopy (XPS) of FeN_4_/C is shown in Figure S7 (Supporting Information), in which the characteristic Fe 2p_3/2_ peak is located at about 710 eV, indicating the existence of Fe^2+^ species in FeN_4_/C.^[^ ^13^ ^]^ The Fourier‐transformed (FT) k^3^‐weighted extended X‐ray absorption fine structure (EXAFS) spectra are shown in Figure 2b. For FeN_4_/C, its FT curve only displays a prominent peak at 1.5 Å, which is generally attributed to the Fe—N first coordination shell.^[^ ^14^ ^]^ Beyond this distance, no obvious FT peaks are observed, especially at the distance of Fe–Fe interaction, indicating that the Fe atoms in FeN_4_/C are atomically dispersed. For Fe NPs/C, a distinct peak corresponding to Fe—Fe bond appears, demonstrating the existence of metallic Fe particles. The quantitative simulation for the EXAFS of FeN_4_/C was performed to obtain the precise chemical configuration around Fe atoms. Figure 2c shows that the experimental FT‐EXAFS curve of the FeN_4_/C has been perfectly reproduced. The fitting results reveal isolated Fe atoms are coordinated with four N atoms at a distance of 1.97 Å. The FeN_4_ structure has been also demonstrated as a more reasonable configuration after the high‐temperature pyrolysis process.^[^ ^8a^ ^]^ The relevant fitting parameters are given in Table S1 (Supporting Information). To further determine the chemical bond for FeN_4_ sites, N 1s XPS was performed, as shown in Figure 2d. The N 1s spectra of N/C could be deconvolved into four peaks with binding energy at 398.4, 399.1, 401, and 404.14 eV, corresponding to pyridinic N, pyrrolic N, graphitic N, and N‐oxide, respectively.^[^ ^15^ ^]^ For FeN_4_/C, the peak assigned to pyridinic N clearly shifts to the higher energy side compared with that of N/C, indicating the pyridinic N bonds with the Fe atoms.^[^ ^16^ ^]^ Thus the local structure of FeN_4_ sites is revealed that the isolated Fe atoms coordinated with four pyridinic N atoms.

**Figure 2:**
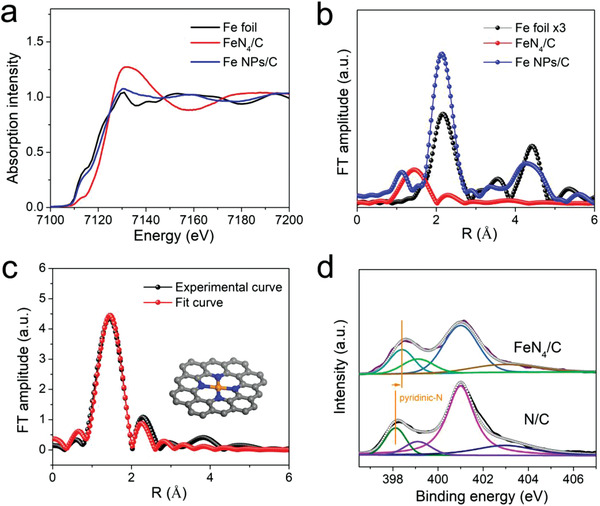
a) Fe K‐edge XANES spectra of Fe foil, FeN_4_/C, and Fe NPs/C. b) FT of the Fe K‐edge EXAFS oscillations of Fe foil, FeN_4_/C, and Fe NPs/C. c) EXAFS fitting curve for FeN_4_/C. Inset is the schematic model of FeN_4_ structure, with Fe in orange, N in blue, and C in gray. d) N 1s XPS spectra of FeN_4_/C and N/C.

The electrocatalytic CO_2_ reduction measurement demonstrates the introduction of isolated FeN_4_ sites in carbon substrate greatly enhances the catalytic performance. The linear sweep voltammetry (LSV) curves (**Figure** **3**a) show that FeN_4_/C gives a much higher catalytic current density than that of N/C and Fe/C, demonstrating the excellent activity of the isolated FeN_4_ sites for CO_2_ reduction. To evaluate the selectivity for CO_2_ reduction, faradaic efficiency (FE) toward CO was measured and presented in Figure 3b. The results show that FeN_4_/C exhibited high conversion efficiency to CO and greatly suppressed the competitive H_2_ evolution reaction (no liquid product was detected, as shown in the ^1^H NMR spectroscopy in Figure S8, Supporting Information), achieving a maximum FE of 93% for CO at −0.6 V. This performance could be comparable with the state‐of‐the‐art catalysts for CO_2_ reduction (Table S2, Supporting Information). In contrast, the N/C and Fe/C only give a maximum FE of 46% and 23%, respectively. The partial current density for CO production of the catalysts is presented in Figure 3c. It is clearly seen that FeN_4_/C shows a much higher catalytic current density of 2.5 mA cm^−2^ at −0.8 V, which is 35 times and 17 times of that for N/C and Fe/C, respectively. This agrees with the electrochemical impedance spectroscopy (EIS) measurement. As presented by the Nyquist plots in Figure 3d, FeN_4_/C has the much smaller charge transfer resistance than that of N/C and Fe/C, correlating to a faster charge‐transfer process for the CO_2_ reduction reaction.^[^ ^17^ ^]^ Good stability of FeN_4_/C is also demonstrated in the 24 h stability test (Figure S9, Supporting Information).

**Figure 3:**
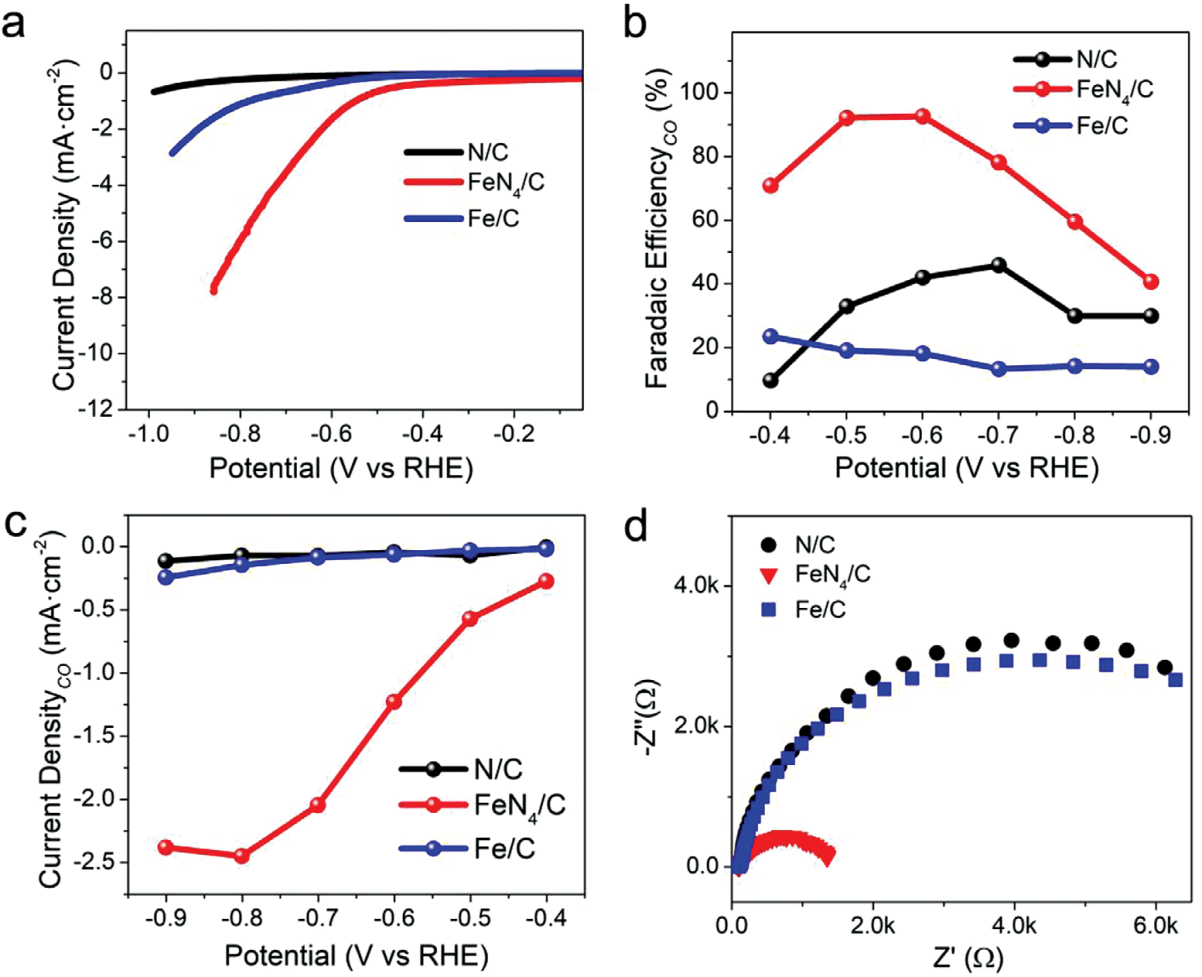
a) LSV curves (iR‐corrected) of N/C, FeN_4_/C, and Fe/C. b) Faradaic efficiencies of N/C, FeN_4_/C, and Fe/C for CO. c) Partial current density for CO production on N/C, FeN_4_/C, and Fe/C. d) Nyquist plots of N/C, FeN_4_/C, and Fe/C.

The influence of the Fe loading on the catalytic activity of FeN_4_/C is shown in Figure S10 (Supporting Information). As seen in the LSV curves, the initial increase of Fe loading leads to a better catalytic activity of CO_2_ reduction, while a further increase of loading to 2.5 wt% (Fe NPs/C) will decrease the catalytic performance. Apparently, the appearance of Fe particles is detrimental to CO_2_ reduction. The FE results in Figure S11 (Supporting Information) also show that the existence of Fe particles suppresses the selectivity for CO_2_ reduction to CO. To compare the intrinsic activity of FeN_4_/C and Fe NPs/C, their electrochemical active surface area (ECSA) was determined by performing cyclic voltammetry test (Figure S12a,b, Supporting Information) to get the double‐layer capacitance (Figure S12c, Supporting Information).^[^ ^18^ ^]^ Fe NPs/C shows a lower ECSA compared with that of FeN_4_/C, indicating a decreased ability for affording the active sites. The intrinsic activity after normalization by the ECSA for the FeN_4_/C and Fe NPs/C is shown in Figure S12d (Supporting Information), in which FeN_4_/C presents an enhanced current density for CO production than that of Fe NPs/C. Based on the catalytic performance, we could see the unique structure and coordination environment endow the isolated FeN_4_ sites with excellent catalytic performance for CO_2_ conversion.

To ravel the high activity of FeN_4_/C for CO_2_ conversion to CO, density functional theory (DFT) calculations were performed. Considering the formation of COOH* is the initial step for the reduction of CO_2_ to CO,^[^ ^19^ ^]^ we first explored the electronic structure of FeN_4_ and N/C sites with adsorbed COOH*. **Figure** **4**a,b present the charge density difference of FeN_4_/C and N/C with COOH* adsorption from the section (two‐dimensional contour map along *z*‐axis) and three‐dimensional view. For FeN_4_ sites, it could be seen that the depletion of electron density appears at Fe site and the electron density accumulation occurs on the C atom of adsorbed COOH*, indicating a charge transfer happens from the Fe site to C atom. While there is no obvious electron interaction between N/C and COOH*. This charge transfer for FeN_4_ sites results in the effective binding strength for the adsorbed intermediate, thereby effectively modulating the energy barrier in CO_2_ reduction process. As shown in Figure 4c, for both N/C and FeN_4_/C, the formation of COOH* is the rate‐limiting step for CO_2_ reduction reaction (detailed energies of adsorbates can be found in Tables S3 and S4, Supporting Information). Benefiting from the charge transfer, the introduction of FeN_4_ sites greatly decreases the barrier for the formation of COOH* compared with that of N/C, thus facilitating the subsequent reduction process, eventually resulting in the enhanced activity for CO production. Different from FeN_4_ structure, Fe nanoparticle shows much strong adsorption for CO*, as shown in Figure S13 (Supporting Information), which restricts the further desorption of CO, and thus leads to relative low selectivity.

**Figure 4:**
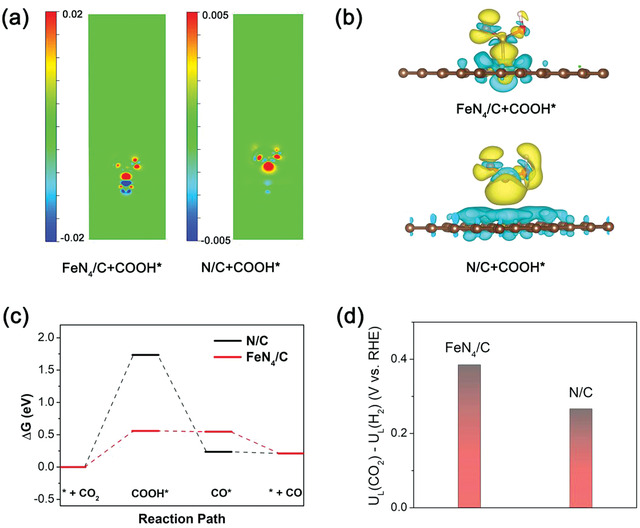
a) Charge density difference of FeN_4_/C and N/C with COOH* adsorption from the section (two‐dimensional contour map along the *z*‐axis) and b) three‐dimensional view. The red (yellow) and blue (turquoise) areas represent electron accumulation and depletion, respectively. c) Calculated free energy diagram for CO_2_ reduction to CO. d) Difference in limiting potentials for CO_2_ reduction and H_2_ evolution.

Considering that H_2_ evolution is the competitive reaction, the difference between thermodynamic limiting potentials for CO_2_ reduction and H_2_ evolution (*U*_L_(CO_2_) – *U*_L_(H_2_)) was also calculated as a reference for the selectivity in CO_2_ reduction reaction. A more positive value would indicate a better selectivity for CO_2_ conversion.^[^ ^20^ ^]^ As shown in Figure 4d, the FeN_4_/C presents a more positive value than that of N/C, demonstrating a higher selectivity for CO_2_ reduction. Based on the DFT analysis, FeN_4_ sites endow the catalyst with a lowered energy barrier for CO_2_ reduction, thus leading to the enhanced activity for CO production.

In conclusion, we have constructed isolated FeN_4_ sites on carbon substrate through a confined pyrolysis strategy using Fe doped g‐C_3_N_4_ as a template. The isolated and well‐defined FeN_4_ sites endow the catalyst with the advantages of both heterogeneous and homogeneous catalysts, showing high activity toward electrocatalytic CO_2_ reduction. Benefiting from the unique structure and coordination environment, the intermediate could be easily formed on the FeN_4_ sites during CO_2_ reduction process, resulting in the greatly improved selectivity for CO production. We anticipate our work will provide valuable guidance for the design of isolated active sites and would inject new vitality to the related electrocatalytic field.

## Conflict of Interest

The authors declare no conflict of interest.

## Supporting information

Supporting Information
