# Supplementary material for: Isolated FeN4 Sites for Efficient Electrocatalytic CO2 Reduction
Source: Adv Sci (Weinh). 2020 Jul 12;7(17):2001545. doi: 10.1002/advs.202001545 (PMC7507046; doi:10.1002/advs.202001545)
Supplement: Supplementary file 1 — Supporting Information [file ADVS-7-2001545-s001.pdf]

Copyright WILEY-VCH Verlag GmbH & Co. KGaA, 69469 Weinheim, Germany, 2020

Supporting Information

Isolated FeN_4 Sites for Efficient Electrocatalytic CO_2 Reduction

*Xiaogang Li, Shibo Xi, Libo Sun, Shuo Dou, Zhenfeng Huang, Tan Su, Xin Wang**

Dr. X. Li, [+] Dr. L. Sun, [+] Dr. S. Dou, Dr. Z. Huang, Prof. X. Wang
School of Chemical and Biomedical Engineering, Nanyang Technological University, 62 Nanyang Drive, Singapore 637459 (Singapore)
E-mail: WangXin@ntu.edu.sg
Dr. S. Xi[+]
Institute of Chemical and Engineering Sciences, A*STAR, Singapore 627833 (Singapore)
Associate Prof. T. Su
Laboratory of Theoretical and Computational Chemistry, Institute of Theoretical Chemistry, Jilin University, Changchun 130012, P. R. China
[+]These authors contributed equally to this work.

Catalyst preparation

Synthesis of g-C_3N_4 :

10 g of urea was placed in a crucible with a lid and heated at 550 °C for 2 h at a rate of 5 °C min^{-1} in muffle furnace without inert atmosphere. The final product was cooled down naturally without further treatment.

Synthesis of N/C:

0.5 g g-C_3N_4 were dispersed in 35 ml 0.3 M glucose solution under sonication for 4 h. Then the above mixture was transferred into a 40 ml Teflon-lined autoclave, sealed and heated at 180 ºC for 10 h. The final product after cleaning and drying was annealed at 1000 °C with a ramp rate of 5 °C min^{-1} in Ar atmosphere for 1 h.

Synthesis of Fe-doped g-C_3N_4:

0.5 g g-C_3N_4 were dispersed in water with addition of $FeCl_3$ (The addition amount of Fe are 0.05, 0.1, 0.25, 0.75 mmol, corresponding to 0.33%, 0.61%, and 1.2 wt% Fe loading in the final FeN_4/C catalysts and 2.5 wt% Fe loading in Fe NPs/C). The solution was stirred for 2 h and followed by freeze-drying. Then the freeze-dried powder was annealed in Ar at 300 °C for 2 h.

Synthesis of FeN_4/C:

0.5 g Fe-doped g-C_3N_4 were dispersed in 35 ml 0.3 M glucose solution under sonication for 4 h and was transferred into a 40 ml Teflon-lined autoclave, sealed and heated at 180 ºC for 10 h. The final product after cleaning and drying was annealed at 1000 °C with a ramp rate of 5 °C min^{-1} in Ar atmosphere for 1 h.

Synthesis of Fe/C

1 g Fe-doped g-C_3N_4 was annealed at 1000 °C with a ramp rate of 5 °C min^{-1} in Ar atmosphere for 1 h.

Characterization

Transmission electron microscopy (TEM) images were taken on JEOL JEM 2100PLUS TEM operated at an acceleration voltage of 200 kV. The X-ray powder diffraction (XRD) patterns were conducted by Bruker D2 Phaser XRD. The high-angle annular dark-field scanning transmission electron microscopy (HAADF-STEM) characterization was performed on a JEOL JEM-ARF200F TEM/STEM with a spherical aberration corrector. Raman spectra were recorded at ambient temperature with Renishaw InVia Reflex Raman Spectrometer excited at 785 nm. The loading of Ni was measured on inductively coupled plasma-atomic emission spectrometer (ICP-AES) on an Optima 7300 DV (PerkinElmer Corporation). X-ray

photoelectron spectra (XPS) were acquired on an ESCALAB MKII with Mg Kα as the excitation source. Fe K-edge XAS data were collected at the beamline 1W1B of the Beijing Synchrotron Radiation Facility (BSRF, Beijing).

Electrochemical measurements

Electrochemical measurements were carried out in H-style cell at an electrochemical station (CHI760E). To prepare the working electrode, 10 µl ink, prepared by dispersing 4 mg sample and 60 µl Nafion solution into 0.94 ml ethanol solution, was loaded onto a glassy carbon electrode with 5 mm diameter. An Ag/AgCl electrode and Pt foil serve as the reference and counter electrode, respectively. LSV test was performed in CO_2-saturated 0.1 M $KHCO_3$ solution with a scan rate of 20 mV/s. The double layer capacitance (C_{dl}) referred the CV results under the potential windows of -0.45 V~-0.55 V (vs Ag/AgCl). The C_{dl} was estimated by plotting the $\Delta j = (j_a - j_c)$ at -0.5 V (vs Ag/AgCl) (where j_c and j_a are the cathodic and anodic current densities, respectively) against the scan rate, in which the slope is as twice as that of C_{dl}. ECSA was calculated by the equation: ECSA = R_fS, where S represented the surface area of the glassy carbon electrode. The roughness factor R_f was estimated from the ratio of C_{dl} for the working electrode and the corresponding smooth electrode (C_s). The value of 40 $\mu F\cdot cm^{-2}$ was used for C_s,[1] that is, $R_f = C_{dl}/(40\ \mu F\cdot cm^{-2})$. Electrochemical impedance spectroscopy (EIS) measurements were carried out by applying an AC voltage with 5 mV amplitude in a frequency range from 100 KHz to 100 mHz. For the faradaic efficiency analysis, gas products during 2-h i-t test were detected by gas chromatograph (Agilent 7890B) and liquid product was characterized by ^1H NMR on Bruker AVANCE AV III 400.

Computational Details

All the spin-polarized first-principle calculations were carried out with the Vienna Ab-initio Simulation Package (VASP) package.[2,3] The projected augmented wave (PAW) was employed to describe the interactions of the core electrons within different atoms.[4] The generalized gradient approximation of Perdew-Burke-Ernzerhof (GGA-PBE) was employed as an exchange-correlation functional.[5] A cutoff energy of 520 eV for the plane wave basis set was used to describe valence electrons of all atoms. The total energy of all the geometry structures were converged to 10^{-5} eV, with the Hellmann-Feynman force converged to 0.02 eV/Å. The models were redefined and established by a 2×4 supercell (a = 8.522 Å, b = 9.840 Å) of graphene with doped iron or nitrogen atoms. The z direction was set perpendicular to the layer plane and the z length was set to be 25 Å to avoid the interaction between adjacent layers. The Brillourin zone was sampled with a gamma-centered grid 4 × 3 × 1 for geometry optimization and 8 × 6 × 1 for self-consistent iteration of single point energy and charge density difference calculation.[6] The DFT + U correction for strong-correlation 3d electrons of transition metal was taken into account, the U-J value of 3.29 is used.[7] As a comparison, the slab model of four-layer iron (100) surface were also constructed with top two layers were allowed to relax. The Van der Waals corrections have been applied in all the structure calculations to account for dispersion interactions.[8,9]

In aqueous electrolytes, the reduction of CO_2 to produce CO could occur through following elementary steps[10]:

$CO_2 + (H^+ + e^-) + * \rightarrow *COOH$

$*COOH + (H^+ + e^-) \rightarrow *CO + H_2O$

$*CO \rightarrow CO + *$

Where * indicates the active sites of the catalyst surface and *COOH and *CO were two key intermediates in CO_2 reduction. The computational hydrogen electrode (CHE) model proposed by Norskov et al.[11] was used to describe the free energy of proton-electron pairs in

the proton-electron transfer steps by using the free energy of hydrogen. The free energies (G)[12] were calculated by correction of electronic energies with including the zero-point energy (ZPE) and the contributions from thermal and entropic terms:

$\Delta G_{DFT} = \Delta E_{elec} + \Delta E_{ZPE} + \int C_p \, dT - T\Delta S$

The data was treated with assistance of vaspkit.1.00 software.[13]

In the calculation of thermodynamic limiting potentials (U_L), $U_L = -\Delta G_0/e$.

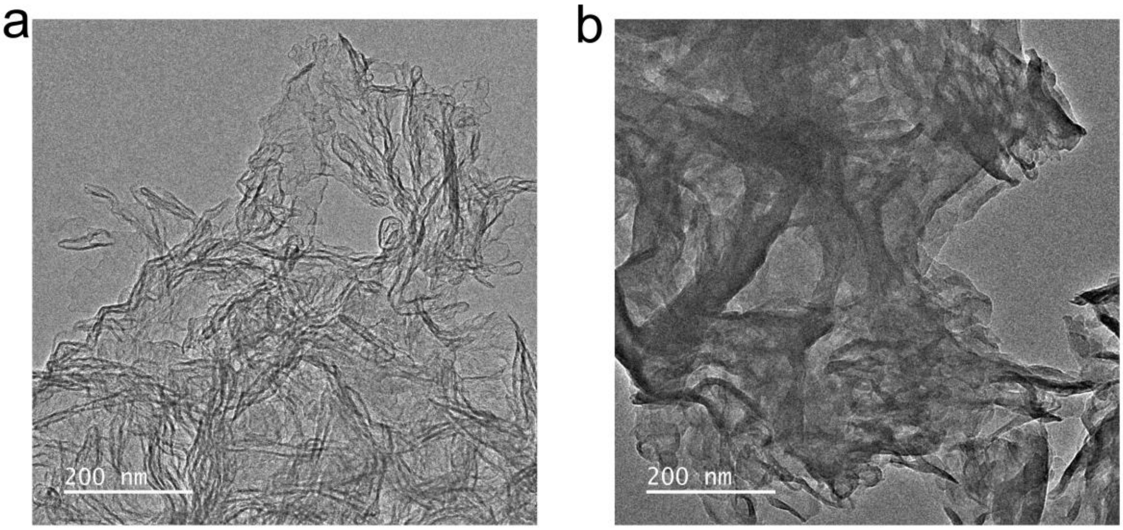

Figure S1. TEM image of N/C and g-C_3N_4.

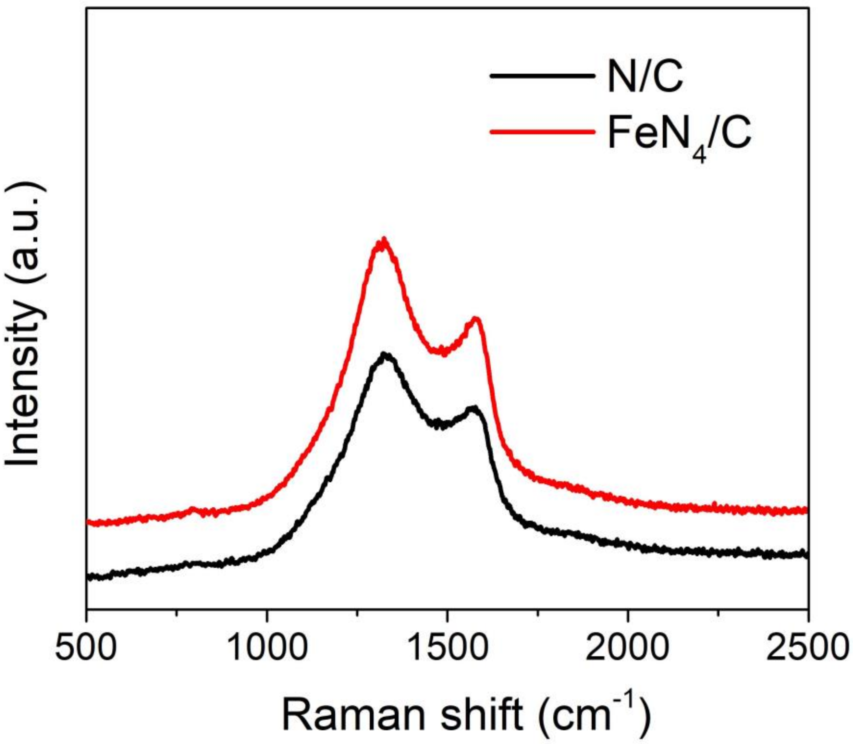

Figure S2. Raman spectra of FeN_4/C and N/C.

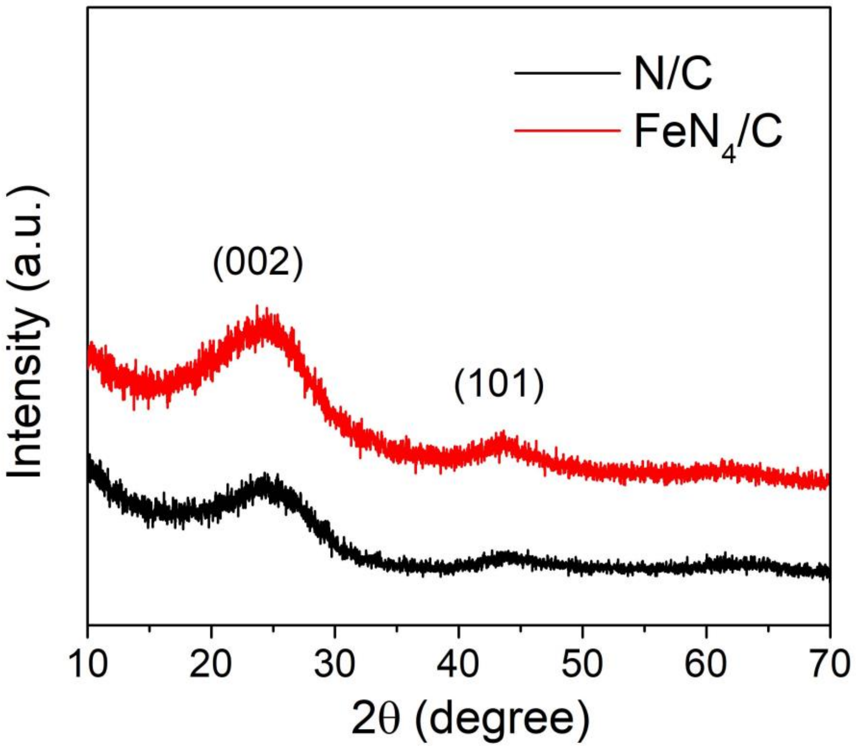

Figure S3. XRD patterns of FeN_4/C and N/C.

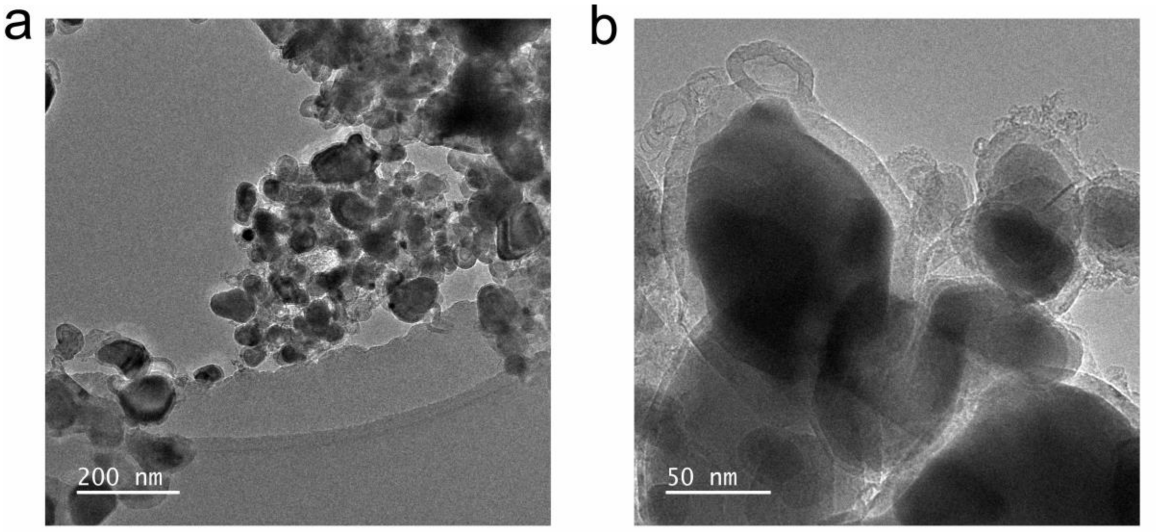

Figure S4. TEM images of Fe/C with low (a) and high (b) resolution.

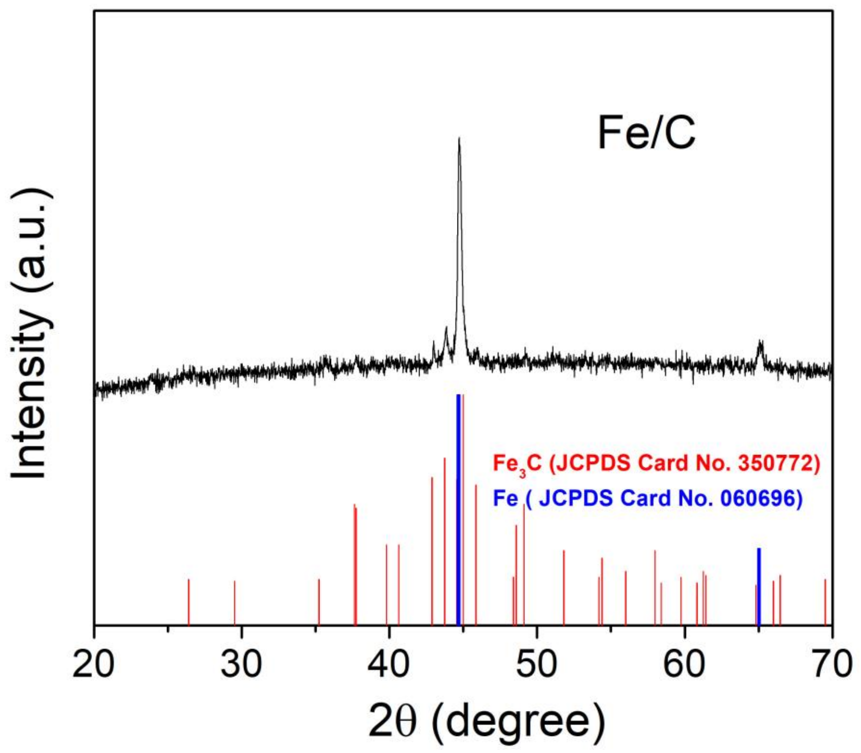

Figure S5. XRD pattern of Fe/C.

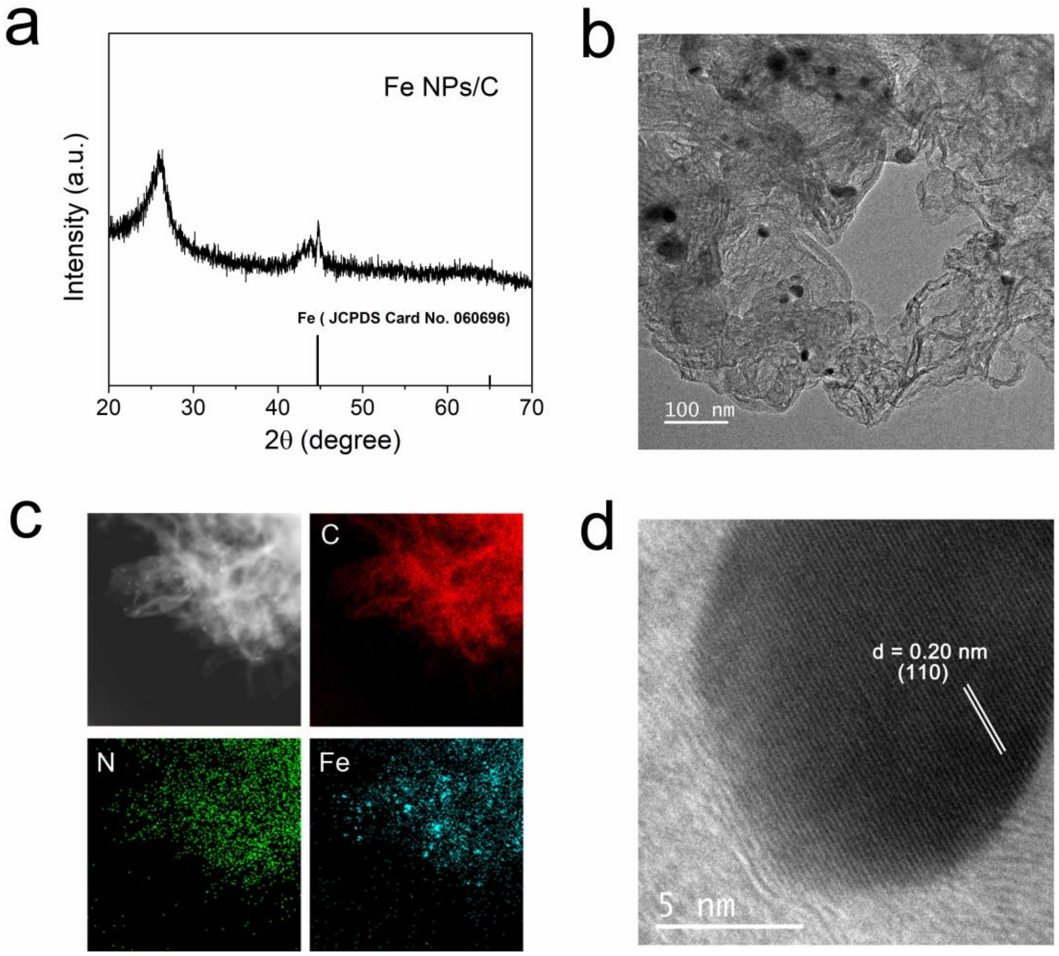

Figure S6. XRD pattern (a), TEM image (b), element mapping image (c) and HRTEM image (d) of Fe NPs/C.

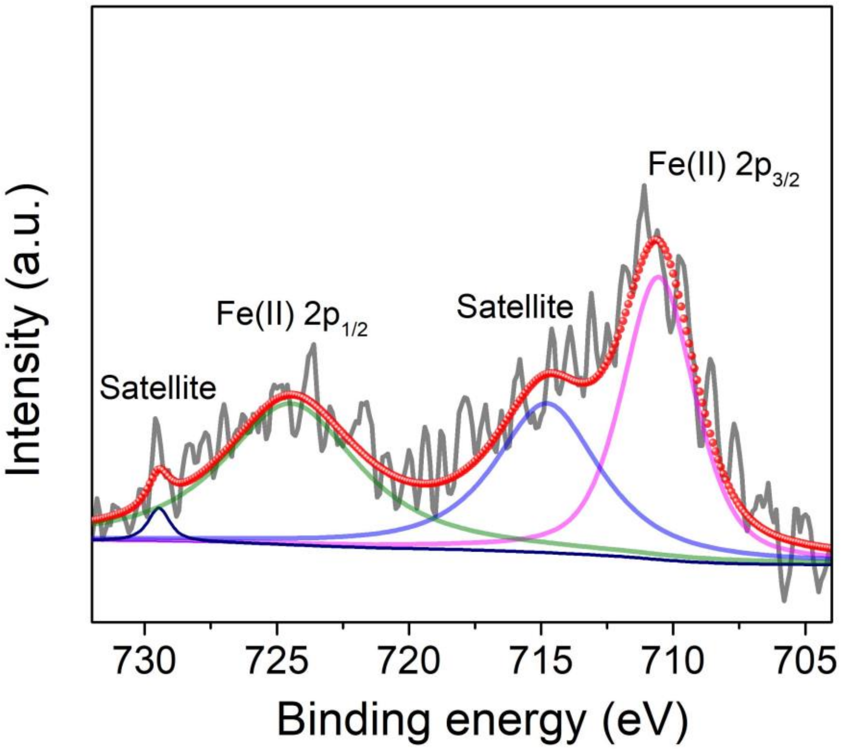

Figure S7. Fe 2p XPS result of FeN_4/C.

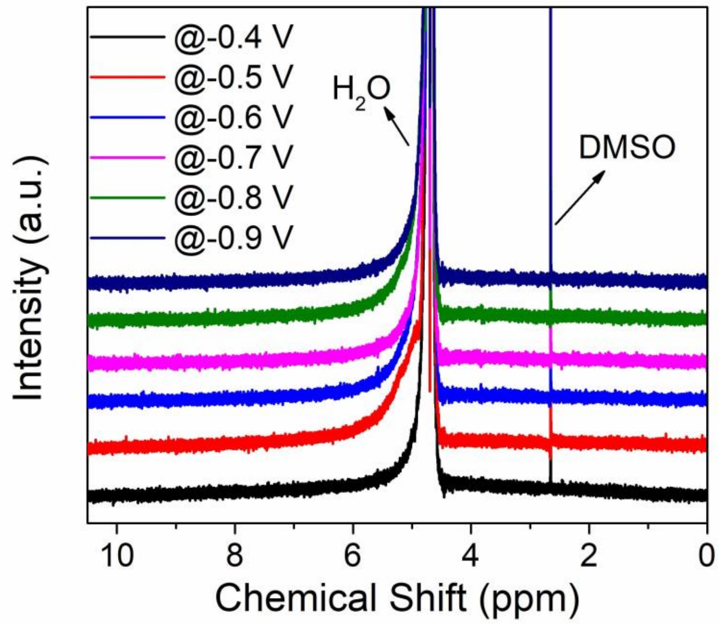

Figure S8. Characterization for the liquid product of FeN_4/C by ^{1}H nuclear magnetic resonance spectroscopy.

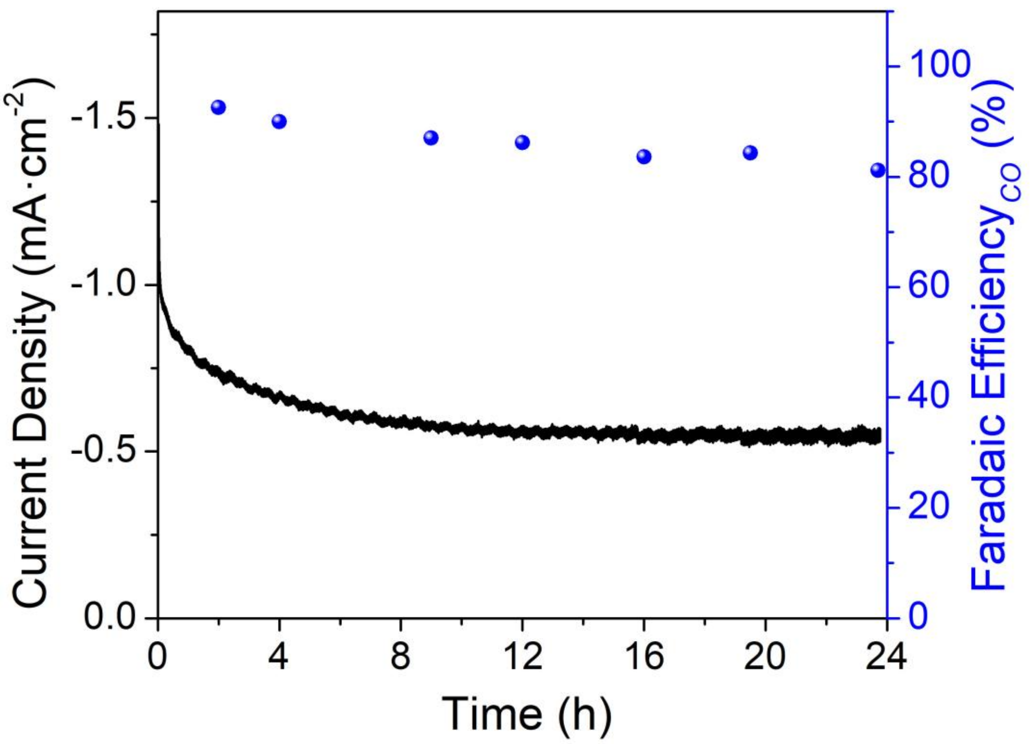

Figure S9. Stability test of FeN_4/C at -0.6 V.

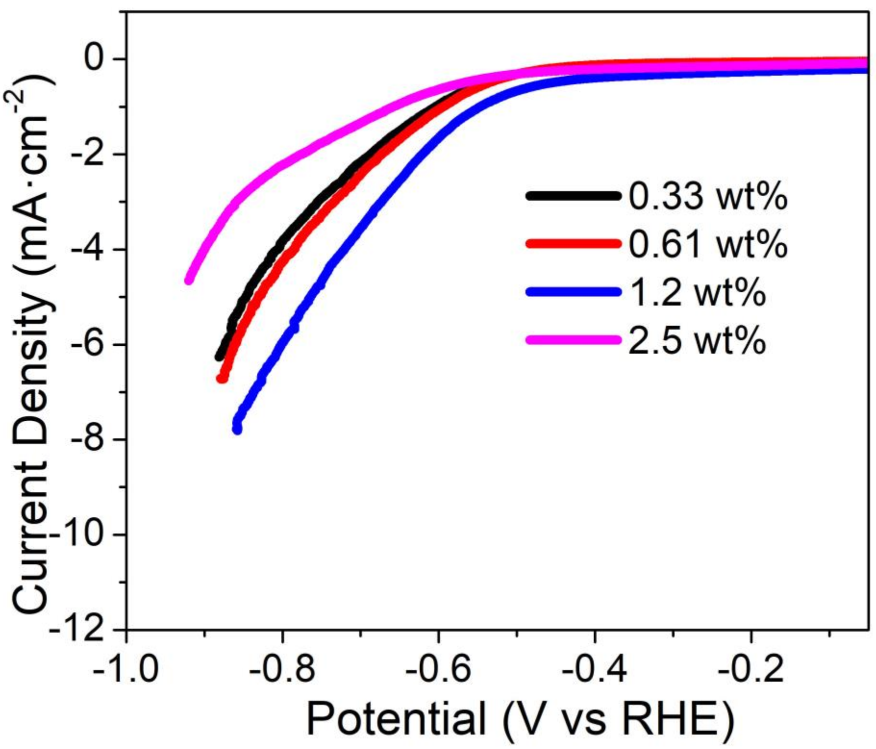

Figure S10. LSV curves (iR-corrected) of FeN_4/C with different Fe loadings.

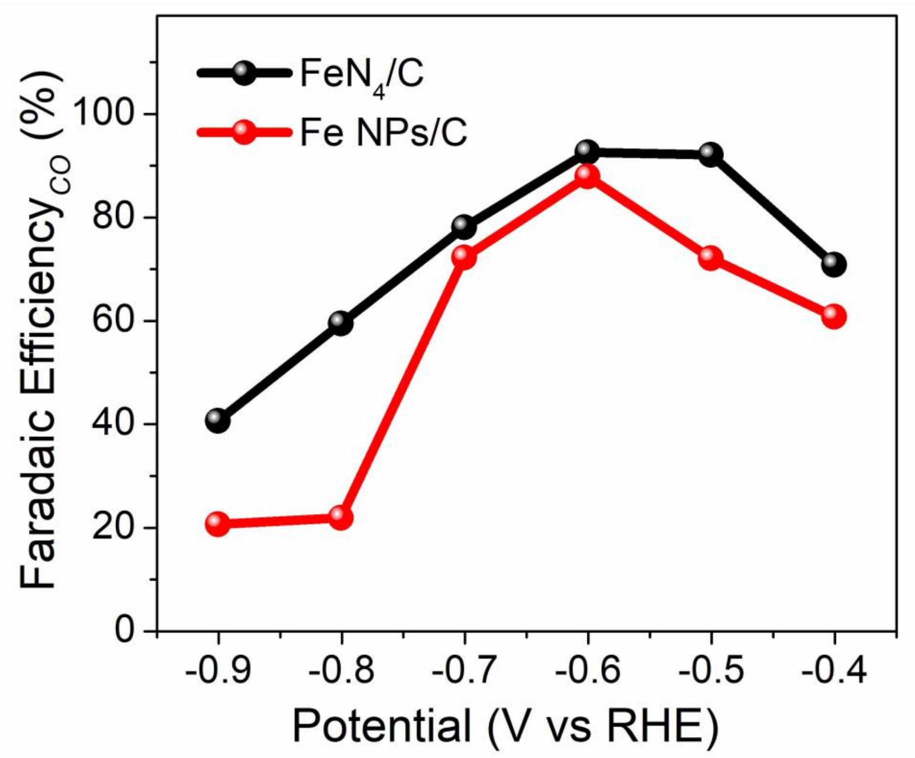

Figure S11. Faradaic efficiencies of FeN_4/C and Fe NPs/C for CO.

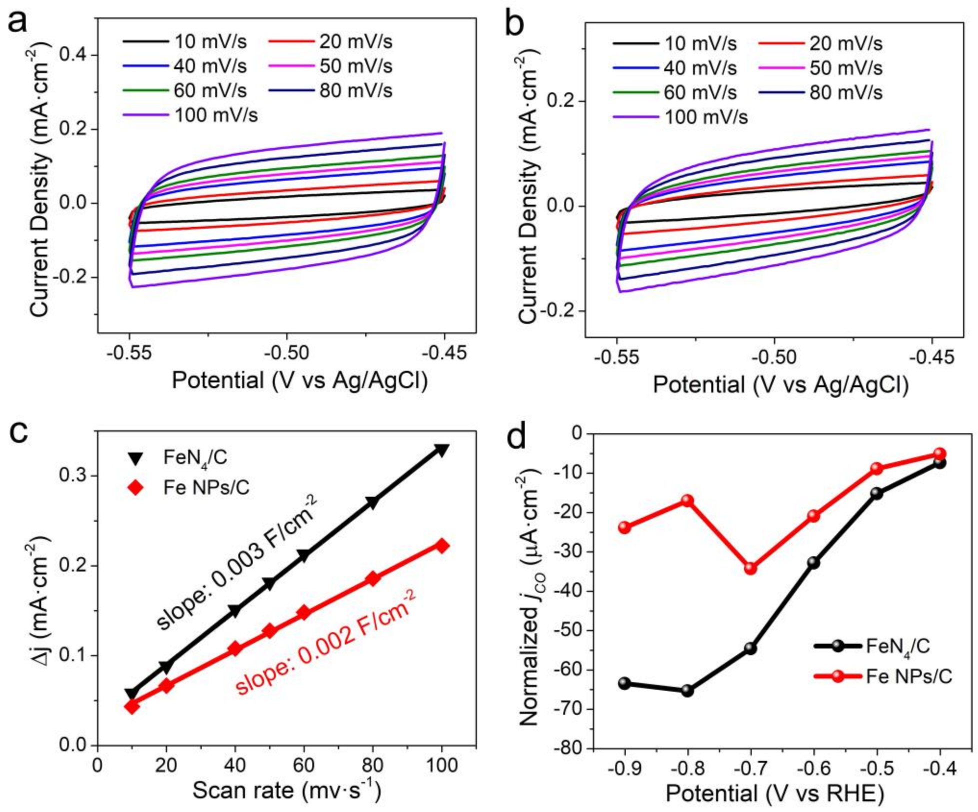

Figure S12. Cyclic voltammograms of FeN_4/C (a) and Fe NPs/C (b). (c) Charging current density differences at -0.5 V vs Ag/AgCl against scan rates for FeN_4/C and Fe NPs/C. (d) ECSA-normalized current densities for CO of FeN_4/C and Fe NPs/C.

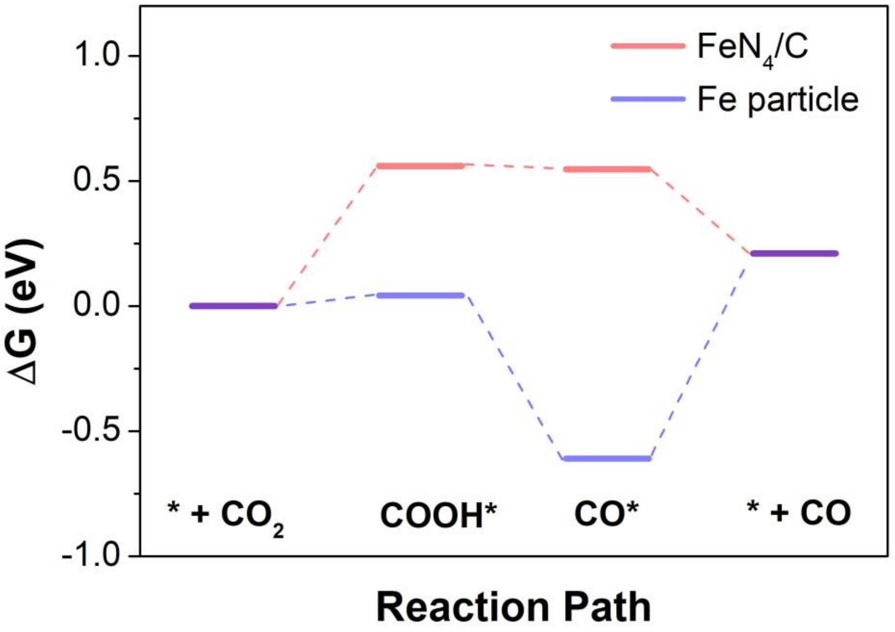

Figure S13. Calculated free energy diagram of FeN_4/C and Fe NPs/C for CO_2 reduction to CO.

Table S1. Fitting results of EXAFS for FeN_4/C by the IFEFFIT code. N is the coordination number. R is the distance between absorber and backscatter atoms. σ^2 is the Debye–Waller factor to account for both thermal and structural disorders. R factor indicates the goodness of the fit.

	pair	*N*	*R* (Å)	σ^2 ($\times10^{-3}$Å^2)	*R factor*
FeN_4/C	Fe-N	4.7 ± 0.6	1.97 ± 0.01	8.6 ± 2.3	0.006

Table S2. A comparison of FeN_4/C with other typical catalysts for the electroreduction of CO_2 to CO.

Catalysts	Electrolyte	Peak potential	FE_{co}	Reference
FeN_4/C	0.1 M $KHCO_3$	-0.6 V	93 %	This work
Co particle @ pyridinic N/C	0.5 M $KHCO_3$	-0.9 V	84 %	Angew. Chem. Int. Ed. 2020, 59, 4914
Ni-N_3-V	0.5 M $KHCO_3$	-0.8 V	94 %	Angew. Chem. Int. Ed. 2020, 59, 1961
NiPor-CTF	0.5 M $KHCO_3$	-0.9 V	97 %	Adv. Funct. Mater. 2019, 29, 1806884.
Isolated Ni/Fe–N Sites	0.5 M $KHCO_3$	-0.7 V	98 %	Angew. Chem. Int. Ed. 2019, 58, 6972
Cobalt Complexes/N Doped Graphene	0.1 M $KHCO_3$	-0.8 V	97 %	Angew. Chem. Int. Ed. 2019, 58, 13532.
FeN_5/C catalyst	0.1 M $KHCO_3$	-0.46 V	97 %	Angew. Chem. Int. Ed. 2019, 58, 14871.
STPyP-Co	0.5 M $KHCO_3$	-0.62 V	96 %	Angew. Chem. Int. Ed. 2019, 58, 12711.
Ni–NG	0.5 M $KHCO_3$	-0.62 V	95 %	Energy Environ. Sci., 2018, 11, 893
Vo-rich ZnO	0.1 M $KHCO_3$	-1.1 V	83%	Angew. Chem. Int. Ed. 2018, 57, 6054.
FePGF	0.1 M KCl	-0.48 V	97 %	Adv. Energy Mater. 2018, 8, 1801280
In/Cu NWs	0.1 M $KHCO_3$	-0.6 V	93 %	ACS Catal. 2018, 8, 6571
Ni−N_4-C	0.5 M $KHCO_3$	-0.81 V	99 %	J. Am. Chem. Soc. 2017, 139, 42, 14889.
Copco-CN/CNT	0.1 M $KHCO_3$	-0.63	98 %	Nat. Commun. 2017, 8, 14675.
Tri-AgNPs	0.1 M $KHCO_3$	-0.856 V	96.8%	J. Am. Chem. Soc. 2017, 139, 6, 2160.
Ordered AuCu NPs	0.1 M $KHCO_3$	-0.77 V	80 %	J. Am. Chem. Soc. 2017, 139, 24, 8329.

Table S3. Total energies of adsorbates. Total energies (eV) of adsorbates from DFT and ZPE and TS contributions to the free energies at the standard conditions.

Catalyst	*Species*	*G (eV)*
FeN_4/C	*	-281.11
	COOH*	-306.49
	CO*	-295.68
	H*	-283.57
N/C	*	-296.20
	COOH*	-320.40
	CO*	-311.08
	H*	-297.60
Fe (100)	*	-243.47164
	COOH*	-270.07722
	CO*	-259.96512
	H*	-246.65621

Table S4. Total energies of small molecules.

Species	*G (eV)*
CO_2	-22.53
CO	-14.91
H_2	-6.82
H_2O (0.035bar)	-14.23

Reference

1. D. Liu, X. Li, S. Chen, H. Yan, C. Wang, C. Wu, Y. A. Haleem, S. Duan, J. Lu, B. Ge, P. M. Ajayan, Y. Luo, J. Jiang, L. Song, *Nat. Energy* **2019**, *4*, 512-518.
2. Käckell,P., Furthmüller,J., Bechstedt, F., Kresse, G. and Hafner, J. Phys. Rev. B. **1996**, *54*, 304-307.
3. Kresse,G. and Furthmüller,J. Comp. Mater. Sci. **1996**, *6*, 15-50.
4. Mortensen,J.J.,Hansen,L. B. and Jacobsen, K. W. Phys. Rev. B. **2005,** *71*, 1-11.
5. Perdew, John P., Burke, K. and Ernzerhof, M. Phys. Rev. Lett. **1996,** *77*, 3865-3868.
6. Monkhorst, H. J. and Pack, J. D. Phys. Rev. B. **1976**, *13*, 5188-5192.
7 Xu, H., Cheng, D., Cao, D. and Zeng, X.C., *Nat. Catal.*, **2018**, *1*, 339-348.
8. Grimme S., Antony J., Ehrlich S., Krieg H., J. Chem. Phys. **2010,** *132*, 154104-154123.
9. Grimme S., Ehrlich S., Goerigk L., J. Comput. Chem., **2011**, *32*, 1456-1465.
10. Wang, S.; Petzold, V.; Tripkovic, V.; Kleis, J.; Howalt, J. G.;Sku´lason, E.; Ferna´ndez, E. M.; Hvolbæk, B.; Jones, G.; Toftelund, A.;Falsig, H.; Björketun, M.; Studt, F.; Abild-Pedersen, F.; Rossmeisl, J.;Nørskov, J. K.; Bligaard, T. Phys. Chem. Chem. Phys. **2011**, *13*20760.
11. Nørskov, J. K.; Rossmeisl, J.; Logadottir, A.; Lindqvist, L.; Kitchin, J. R.; Bligaard, T.; Jonsson, H. J. Phy. Chem. B **2004**, *108*, 17886-17892.
12. Peterson, A. A.; Abild-Pedersen, F.; Studt, F.; Rossmeisl, J.; Nørskov, J. K. Energy. Environ. Sci. **2010,** *3*, 1311-1315.
13. Wang V, Xu N, Liu JC, Tang G, Geng WT. arXiv preprint, **2019**, Aug 22, arXiv:1908.08269.
